# A Simplified CNN Classification Method for MI-EEG via the Electrode Pairs Signals

**DOI:** 10.3389/fnhum.2020.00338

**Published:** 2020-09-15

**Authors:** Xiangmin Lun, Zhenglin Yu, Tao Chen, Fang Wang, Yimin Hou

**Affiliations:** ^1^College of Mechanical and Electric Engineering, Changchun University of Science and Technology, Changchun, China; ^2^School of Automation Engineering, Northeast Electric Power University, Jilin, China

**Keywords:** brain computer interface (BCI), electroencephalography (EEG), electrode pairs, motor imagery (MI), convolutional neural network (CNN)

## Abstract

A brain-computer interface (BCI) based on electroencephalography (EEG) can provide independent information exchange and control channels for the brain and the outside world. However, EEG signals come from multiple electrodes, the data of which can generate multiple features. How to select electrodes and features to improve classification performance has become an urgent problem to be solved. This paper proposes a deep convolutional neural network (CNN) structure with separated temporal and spatial filters, which selects the raw EEG signals of the electrode pairs over the motor cortex region as hybrid samples without any preprocessing or artificial feature extraction operations. In the proposed structure, a 5-layer CNN has been applied to learn EEG features, a 4-layer max pooling has been used to reduce dimensionality, and a fully-connected (FC) layer has been utilized for classification. Dropout and batch normalization are also employed to reduce the risk of overfitting. In the experiment, the 4 s EEG data of 10, 20, 60, and 100 subjects from the Physionet database are used as the data source, and the motor imaginations (MI) tasks are divided into four types: left fist, right fist, both fists, and both feet. The results indicate that the global averaged accuracy on group-level classification can reach 97.28%, the area under the receiver operating characteristic (ROC) curve stands out at 0.997, and the electrode pair with the highest accuracy on 10 subjects dataset is FC3-FC4, with 98.61%. The research results also show that this CNN classification method with minimal (2) electrode can obtain high accuracy, which is an advantage over other methods on the same database. This proposed approach provides a new idea for simplifying the design of BCI systems, and accelerates the process of clinical application.

## 1. Introduction

Motor imagery electroencephalography (MI-EEG) is a self-regulated EEG without an external stimulus, which can be detected by electrodes. It was suggested in a literature survey that MI is consistent with changes caused by actual exercise in the motor cortex region (Jenson et al., 2019; Kwon et al., 2019).

A brain-computer interface (BCI) is a communication channel between the brain and the outside world, and various types of thinking activities in the brain can be detected through EEG (Atum et al., 2019; Mebarkia and Reffad, 2019; Meziani et al., 2019). The application of BCI in rehabilitation training can help normal thinking patients with paralysis of the neuromuscular system interact with the outside world (Leeb et al., 2015; Rupp et al., 2015; Müller-Putz et al., 2017; Wang L. et al., 2019). In addition, EEG studies were conducted on the control of an intelligent wheelchair (Zhang et al., 2016; Pinheiro et al., 2018), robotic arm (Meng et al., 2016), and other external devices (He et al., 2015; Edelman et al., 2019). A major challenge of the BCI is to interpret movement intention from brain activity. Efficient neural decoding algorithm can significantly improve the decoding accuracy, which can improving the performance of BCI. The low signal-to-noise ratio of EEG leads to low classification accuracy. Therefore, effective feature extraction and classification methods have become an important research topic of MI-EEG (Li et al., 2019). Commonly used feature extraction algorithms include wavelet transform (WT) (Xu et al., 2018), common spatial patterns (CSP) (Kumar et al., 2016), variations of CSP (Kim et al., 2016; Sakhavi and Guan, 2017), empirical mode decomposition (EMD) (Kevric and Subasi, 2017), and so on.

Deep learning (DL) has attracted attention in many areas for its superior performance. DL can effectively deal with nonlinear and non-stationary data, and learn underlying features from signals. Some deep learning methods are employed for the classification of EEG signals (Cecotti and Graser, 2010; Bashivan et al., 2015; Corley and Huang, 2018). Convolutional neural networks (CNNs) have been widely used in MI-EEG classification on account of their ability to learn features from local receptive fields. Because the trained detector can be used to detect abstract features by convolutional layer repetition, CNNs are suitable for complex EEG recognition tasks, and have achieved good results and been widely used by many scholars (Amin et al., 2019; Hou et al., 2019; Jaoude et al., 2020; Zhang et al., 2020).

Preprocessing raw EEG signals can improve the signal-to-noise ratio of EEG and the classification accuracy, but it is not necessary. CNNs are the biologically inspired variants of multilayer perceptrons designed to use minimal preprocessing (LeCun et al., 1998). For example, Dose et al. (2018) and Tang et al. (2017) used CNN to directly classify raw EEG signals. Shen et al. (2017) combined RNNs with CNN to enhance the feature representation and classification capabilities of raw MI-EEG, which was inspired by speech recognition and natural language processing. Schirrmeister et al. (2017) established a deeper layer of the neural network to decode imagine or perform tasks from raw EEG signals. Hajinoroozi et al. (2016) proposed an improved CNN with raw EEG signals to predict a driver's cognitive state related to driving performance, which achieved good results. It can be seen that using the original signals can also obtain a good MI-EEG classification effect. CNNs can take multidimensional data as input directly, avoiding the complicated artificial feature extraction process, which can extract distinctive feature information.

The number of electrodes affects the classification accuracy. In general, higher accuracy can be achieved with more electrodes based on the comparison results of Yang et al. (2015) and Cecotti and Graser (2010). Karácsony et al. (2019) further explained that increasing the number of electrodes can improve the accuracy of classification and recognition without changing the data set and classification method. However, the increase of the number of electrodes will increase the complexity of BCI systems. Although some BCIs have better recognition accuracy, the system structures are complex (Chaudhary et al., 2020; Tang et al., 2020).

In this paper, we proposed a CNN architecture with separated temporal and spatial filters, which classifies the raw MI-EEG signals of the left and right brain symmetric electrodes, without any preprocessing and artificial feature extraction operations. It has a 5-layer CNN, in which four layers are convoluted along the temporal axis, and the other layer is convoluted along the spatial axis. It uses 4-layer max pooling to reduce dimensionality, and a fully-connected (FC) layer to classify. Dropout and batch normalization are used to reduce the risk of overfitting.

CNNs have made remarkable achievements in the field of image classification. Multi-channel EEG data are also two-dimensional, but the time and channel of EEG have different units. Different from other CNN methods used EEG data as images for classification, our method uses separate time and space filters, and focuses on the detection of time-related features in EEG signals, which helps to improve the accuracy.

Deep learning usually provides better classification performance by increasing the size of training data. On the basis of Physionet database, we also set up a hybrid dataset including 9 pairs of electrode samples of 100 subjects. Each sample only contains information from a single pair of electrodes from a single subject. So the dimension of a sample and the processing difficulty are reduced.

The remainder of this paper is organized as follows: section 2 briefly introduces the dataset. In section 3, the CNN theory, construction and classification are described. Details of the experimental results and analysis are discussed in section 4. Finally, section 5 concludes this paper.

## 2. Materials and Methods

### 2.1. The Framework

**Figure 1:**
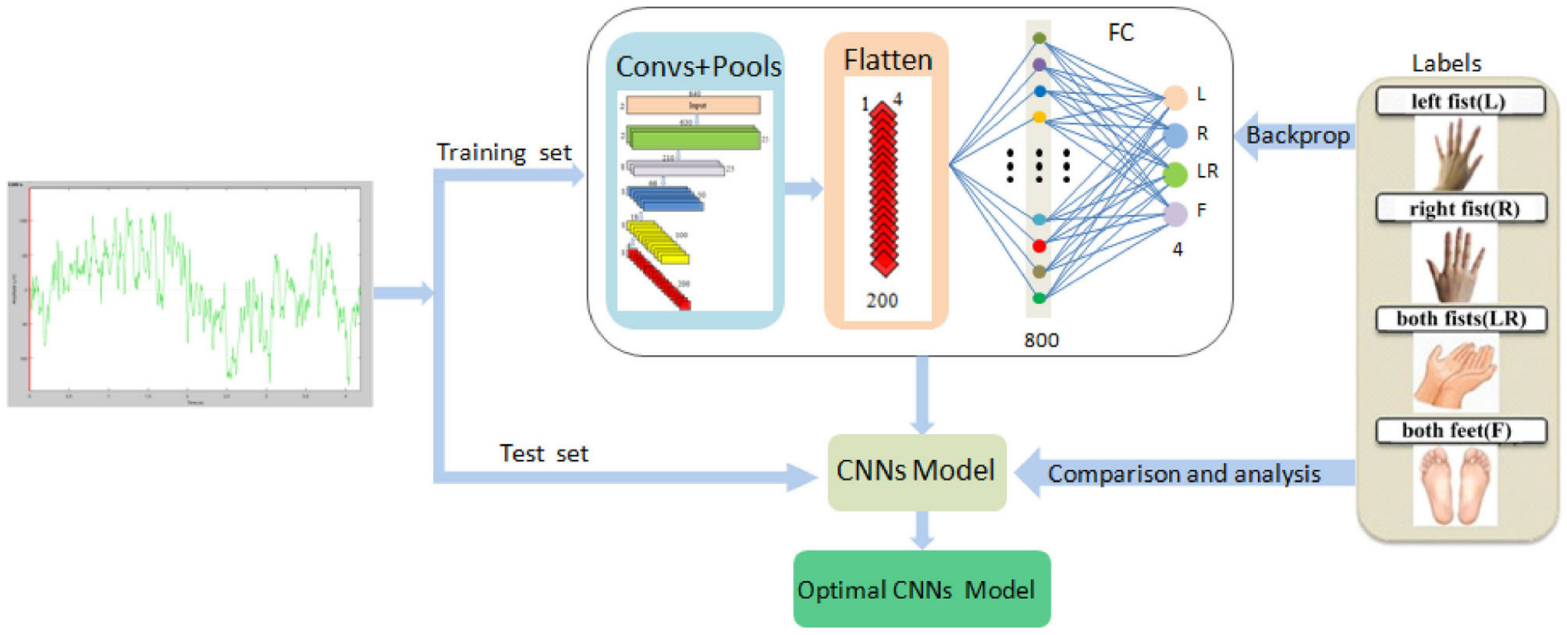
The system framework of the simplified CNN classification method for MI-EEG via the electrode pairs signals, including extraction of raw signals of nine pairs of symmetric electrodes, 5-layer CNN, 4-layer max pooling, and the FC layer.

The system framework of the proposed method is demonstrated in Figure 1.

1. We downloaded the data of each subject, shuffled randomly according to the trial, and then divided the data into 10 pieces. For each subject's data, our operation process was like this.
2. We took one piece as the test set and the other nine as the training set. In the test set and training set, we pieced together the data from multiple subjects. The MI-EEG raw signals of nine pairs of symmetrical electrodes over the motor cortex region were extracted from each trial, and the signals of each pair constituted a sample.
3. We trained our proposed CNN model using the training set. The 5-layer CNN learned EEG features, and the 4-layer max pooling reduced the dimensions. The FC layer divided MI into four types: left fist, right fist, both fists, and both feet. Then, comparing with the four types of labels, the optimal training model can be obtained. Finally, we verified the validity of the model on the test set.
4. Adopting 10-fold cross validation, model training and testing were carried out 10 times, thus providing us with 10 results. Their average values are used as the global average accuracy.

### 2.2. Dataset

This paper used the Physionet MI-EEG database, which was recorded by the developers of the BCI2000 system (Goldberger et al., 2000; Schalk et al., 2004). According to the international 10-10 system (excluding electrodes NZ, F9, F10, FT9, FT10, A1, A2, TP9, TP10, P9, and P10), the original data are extracted from 64 electrodes, including 4 MI tasks. The database contains more than 1,500 one-minute and two-minute EEG records from 109 different subjects, with a sampling frequency of 160 Hz.

EEG data acquisition typically uses 32 or 64 electrodes. There are many reasons for reducing the number of electrodes used (Tam et al., 2011). First, fewer electrodes can save more time on preparation for electrode placement. Second, fewer electrodes will reduce the cost of acquisition hardware. Third, and most importantly, when running the BCI systems, the overfitting risk of classifiers and spatial filters will increase with the number of irrelevant electrodes.

**Figure 2:**
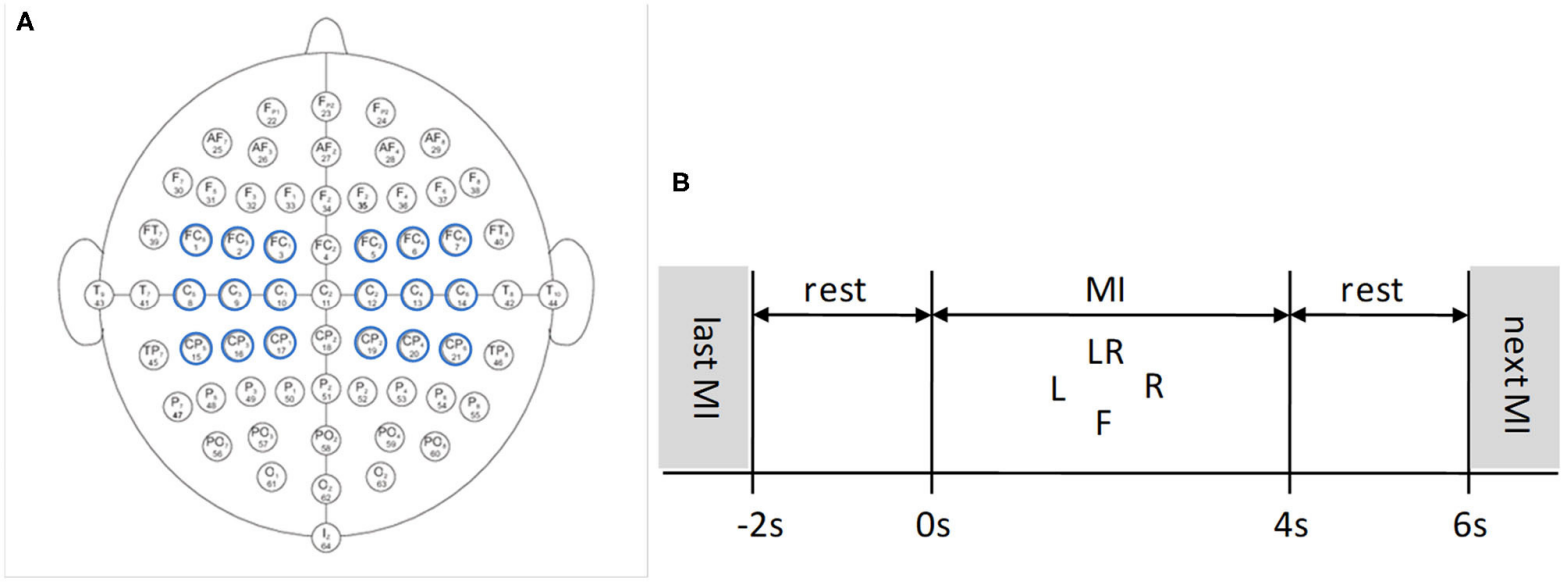
EEG 64 Electrode placement and timing diagram for the Physionet MI-EEG database according to the international 10-10 system. **(A)** Nine pairs of symmetrical electrodes over the motor cortex region are selected. **(B)** 6 s timing diagram of the trial.

It is important to select proper electrodes and their locations in BCI systems. Fewer electrodes but incorrect locations may lose important information, while too many electrodes may produce redundant information, thereby reducing system performance. Therefore, electrode selection is of great significance for EEG analysis. In this paper, nine pairs of symmetrical electrodes (FC5-FC6, FC3-FC4, FC1-FC2; C5-C6, C3-C4, C1-C2; CP5-CP6, CP3-CP4, CP1-CP2) over the motor cortex region were selected as the research objects, which are displayed in Figure 2A.

Each subject conducted four MI tasks: left fist, right fist, both fists, and both feet, which are called T1, T2, T3, and T4, and 21 trials were performed for each MI task. The timing diagram of the trial is shown in Figure 2B. The trial start time is *t* = −2 s, the subject relaxes for 2 s. At *t* = 0 s, the target appears on the screen:

1. L indicates motor imagination of opening and closing left fist;
2. R indicates motor imagination of opening and closing right fist;
3. LR indicates motor imagination of opening and closing both fists;
4. F indicates motor imagination of opening and closing both feet.

The subject was cued to execute corresponding MI task for 4 s. At *t* = 4 s, the target disappeares, and this trial finished. After 2 s rest, a new trial begins (Dose et al., 2018).

Because the motor imagination were performed around 4 s each time, and the sampling frequency is 160 Hz, then the effective data size of each electrode per trial is 640. A sample contains a pair of symmetrical electrodes, and their data are combined in series, so the data size of a sample is 1,280.

Each subject carried out 21 trials on each MI task, a total of 84 trials. In this paper, 10-fold cross validation was carried out on the datasets. We divided all trials of a subject into 10 parts. For each task class, we used 2 trials for test, and the rest for training. Therefore, there are 8 trials in the test set, and 76 trials in the training set. There are 840 trials in 10 subjects dataset (S1~S10), 760 for training and 80 for testing. There are 1,680 trials in 20 subjects dataset (S1~S20), 1,520 for training and 160 for testing. There are 5,040 trials in 60 subjects dataset (S1~S60), 4,560 for training and 480 for testing. There are 8,400 trials in 100 subjects dataset (S1~S100), 7,600 for training, and 800 for testing. In addition, we extracted 9 samples in each trial. Ten subjects dataset with 7,560 samples, 20 subjects dataset with 15,120 samples, 60 subjects dataset with 45,360 samples, and 100 subjects dataset with 75,600 samples were selected for model training and generalization performance verification.

### 2.3. CNN Theory and Structure

#### 2.3.1. CNN Theory

CNN structure can imitate the complex cerebral cortex of the human brain. It only relies on a large training dataset to train a complex model, which uses backpropagation and gradient descent optimization algorithm to learn features, and uses a series of filtering, normalization, and nonlinear activation operations to extract features (Wu et al., 2019; Mohseni et al., 2020).

Each convolutional layer in CNNs consists of multiple convolutional kernels of the same size for feature extraction. Each kernel is a two-dimensional matrix with weights. The value of each neuron in the convolutional layer is the result obtained by multiplying the data of the previous input layer with a convolution kernel, and then adding the corresponding offset. When performing the feature extraction operation, the kernel sequentially scans the input data of the upper layer according to a certain step and mode setting. In addition, the kernels and the data in the previous layer are dot multiplied to obtain the convolution subgraph (Zhang X. et al., 2019; Zheng et al., 2020).

In the operation of the convolutional layer, two important characteristics of local connection and weight sharing are used (Dai et al., 2019; Sun et al., 2019). The local connection is similar to the local receptive area. It is mainly used to extract features with appropriate granularity and reduce the number of CNN parameters. Weight sharing means that all neurons in the same convolution subgraph have the same weight and bias value, which can reduce the number of network parameters, the amount of calculation and the risk of overfitting (Acharya et al., 2018; Podmore et al., 2019; Zhang X. et al., 2019).

The mathematical expression of the convolutional layer is:

(1) $$\begin{array}{l} y_{mn}=f\left(\overset{J-1}{\underset{j=0}{\sum }}\overset{I-1}{\underset{i=0}{\sum }}x_{m+i,n+j}w_{ij}+b\right) \end{array}$$

where *x* is the input two-dimensional data; *y* is the output of *M* × *N*; 0 ≤ *m* ≤ *M*, 0 ≤ *n* ≤ *N*; *w* is the convolutional kernel with *J* × *I*; *b* is the bias; and *f* is the activation function.

CNNs use the multidimensional original signals as the input of the network, and rely on the backpropagation learning algorithm to turn the hidden layers into suitable feature extractors so as to avoid the complex artificial feature extraction process. CNNs are suitable for signals such as EEG that change greatly over time (Zhang Y. et al., 2019; Zuo et al., 2019).

#### 2.3.2. CNN Structure

We selected 10 subjects as the dataset, with a total of 7,560 samples, in which the training set was 6,840, and the test set was 720. We performed a series of experiments to determine the number of layers and their parameters in the structure. Leaky ReLU (Dose et al., 2018; Macdo et al., 2019) was chosen as the activation function to avoid the vanishing gradient problem. The optimizer adopted the Adam algorithm (Dose et al., 2018; Chang et al., 2019), which updated the weights and bias through the backpropagation algorithm, and the learning rate was 1 × 10^−5^.

In the experiments, each network structure was repeated 10 times, and the number of iterations was 2,000 each time. Finally, we have identified 5-layer CNN and 4-layer max pooling. This model also used dropout and batch normalization to reduce the risk of overfitting.

The selected CNN architecture is shown in Table 1: the first layer is the input layer; the second, third, fifth, seventh, and ninth layers are the convolutional layers; the fourth, sixth, eighth, and tenth layers are the max pooling layers; and the eleventh layer is the FC layer.

**Table 1:** Proposed CNN architecture.

**Layer**	**Type**	**Size**	**Kernel size**	**Stride**	**Padding**
L1	Input	640 × 2			
L2	Convolution1	(1, 630, 2, 25)	[11, 1, 1, 25]	[1, 1, 1, 1]	VALID
	Activation			
	Spatial dropout				
L3	Convolution2	(1, 630, 1, 25)	[1, 2, 25, 25]	[1, 1, 1, 1]	VALID
	Batch				
	Normalization				
	Activation				
L4	Max-Pooling1	(1, 210, 1, 25)	[1, 3, 1, 1]	[1, 3, 1, 1]	VALID
L5	Convolution3	(1, 200, 1, 50)	[11, 1, 25, 50]	[1, 1, 1, 1]	VALID
	Activation				
	Spatial dropout				
L6	Max-Pooling2	(1, 66, 1, 50)	[1, 3, 1, 1]	[1, 3, 1, 1]	VALID
L7	Convolution4	(1, 56, 1, 100)	[11, 1, 50, 100]	[1, 1, 1, 1]	VALID
	Batch normalization				
	Activation				
	Spatial dropout				
L8	Max-Pooling3	(1, 18, 1, 100)	[1, 3, 1, 1]	[1, 3, 1, 1]	VALID
L9	Convolution5	(1, 8, 1, 200)	[11, 1, 100, 200]	[1, 1, 1, 1]	VALID
	Batch normalization				
	Activation				
L10	Max-Pooling4	(1, 4, 1, 200)	[1, 2, 1, 1]	[1, 2, 1, 1]	VALID
L11	Flatten	800			
	Fully-connected	4			

The input data format of CNN is: *T* × *N*, where *T* refers to the sampling amount of each channel and *N* is the number of electrodes used. In this paper, *T* = 640, *N* = 2.

**Figure 3:**
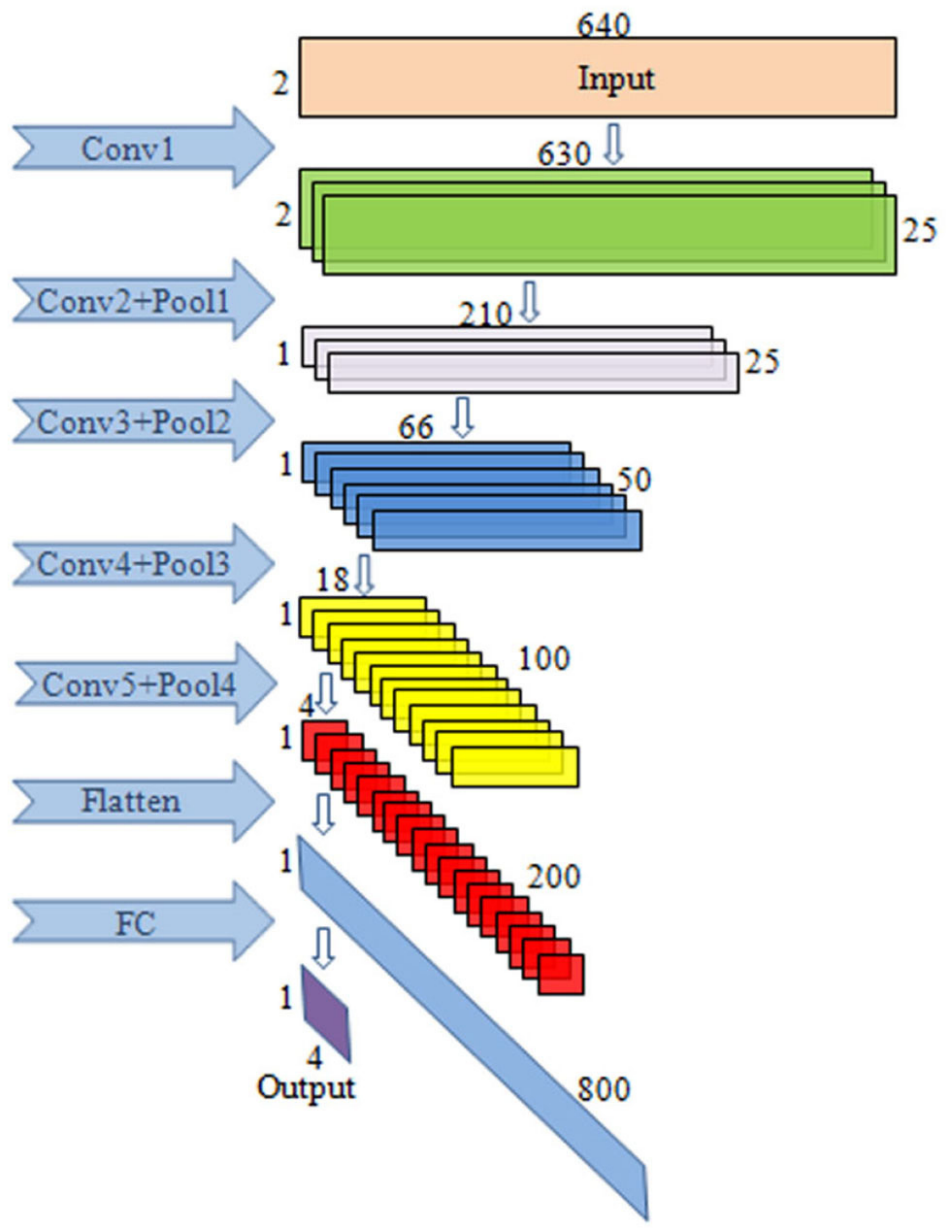
The illustration of the CNN block diagram. The model consists of five convolutional layers, four max pooling layers as well as the FC layers.

The block diagram of CNN is given in Figure 3. This paper mainly uses a one-dimensional convolution, which is helpful for extracting important local features between adjacent element values of the feature vector (Schirrmeister et al., 2017). In convolutional layer 1, one-dimensional convolution in the direction of the temporal axis is carried out, with 25 kernels of [11, 1, 1, 25]. After convolution, the data size becomes (1, 630, 2, 25), and 25 is the channel. In convolutional layer 2, convolution is performed along the spatial axis. The size of kernels is [1, 2, 25, 25], the first 25 is the channel, and the last 25 is the number of kernels. After convolution, the size becomes (1, 630, 1, 25). In pooling layer 1, max pooling is carried out with the core of [1, 3, 1, 1], the stride of [1, 3, 1, 1], and the output size of (1, 210, 1, 25). In convolutional layer 3, convolution is conducted along the temporal axis, and there are 50 kernels with a size of [11, 1, 25, 50]. After convolution, the data size becomes (1, 200, 1, 50). The parameters of pooling layer 2 are the same as those of pooling layer 1, and the output size is (1, 66, 1, 50). In convolutional layer 4, there are 100 kernels with the sizes of [11, 1, 50, 100]. After convolution carried out along the temporal axis, the data size becomes (1, 56, 1, 100). In pooling layer 3, the parameters are the same as above, and the output size is (1, 18, 1, 100). In convolutional layer 5, convolution is performed along the temporal axis. There are 200 kernels with the size of [11, 1, 100, 200], and the data size after convolution becomes (1, 8, 1, 200). In pooling layer 4, max pooling is performed with the core of [1, 2, 1, 1], the stride of [1, 2, 1, 1], and the output size of (1, 4, 1, 200).

The essence of the pooling operation is downsampling. We chose the max pooling, which is realized by taking the maximum value of the features in the neighborhood. It can suppress the phenomenon that the network parameter error causes the shift of the estimated mean value and extract the feature information better.

After feature extraction, the FC layer is applied to enhance the nonlinear mapping capability of the network. It perceives the global information and aggregates the local features learned from the convolutional layer to form the global features for classification. Each neuron in this layer is connected to all neurons in the previous layer, and there is no connection between neurons in the same layer. The formula is

(2) $$\begin{array}{l} y_{j}^{\left(l\right)}=f\left(\overset{n}{\underset{i=1}{\sum }}x_{i}^{\left(l-1\right)}\times w_{ji}^{\left(l\right)}+b^{\left(l\right)}\right) \end{array}$$

where *n* is the number of neurons in the previous layer, *l* is the current layer, $w_{ji}^{\left(l\right)}$ is the connection weight of neurons *j* in this layer and neurons *i* in the previous layer, *b*^(*l*)^ is the bias of neurons *j*, and *f* is the activation function.

The output of the FC layer is generated by a softmax layer, which contains four neurons [*y*^1^, *y*^2^, *y*^3^, *y*^4^], representing the four categories. It maps the output of multiple neurons to the (0, 1) interval, which can be considered as the probability of multi-classification. The formula is as follows:

(3) $$\begin{array}{l} Y^{i}=argmax\left(\frac{e^{y^{i}}}{\sum_{i=1}^{4}e^{y^{i}}}\right) \end{array}$$

In this paper, all activation functions of the networks adopted the leaky ReLU function:

(4) $$\begin{array}{l} f(x)=\{\begin{array}{cc} x, & if\;x>0\\ 0.01x, & otherwise \end{array} \end{array}$$

We used the Adam algorithm as the optimizer to minimize the loss function and update the weight and bias through a backpropagation algorithm. It is a stochastic gradient descent (SGD) algorithm based on the adaptive learning rate of the first-order and second-order moments of the gradient average. This method usually speeds up the convergence of the model and is more robust in the presence of noise and sparse gradients.

The proposed CNN architecture includes the spatial dropout, and the batch normalization (BN) algorithms to improve classification accuracy. Dropout refers to the “temporarily discarding” some neuron nodes with a certain probability during the training of a deep network. For any neuron, each training is optimized together with a randomly selected set of different neurons. This process weakens the joint adaptability among all neurons, reduces the risk of overfitting, and enhances the generalization ability (Srivastava et al., 2014).

The forward propagation formula corresponding to the original network is

(5) $$\begin{array}{l} z_{i}^{\left(l+1\right)}=w_{i}^{\left(l+1\right)}y^{\left(l\right)}+b_{i}^{\left(l+1\right)} \end{array}$$

(6) $$\begin{array}{l} y_{i}^{\left(l+1\right)}=f\left(z_{i}^{\left(l+1\right)}\right) \end{array}$$

After applying dropout, the forward propagation formula becomes:

(7) $$\begin{array}{l} r_{j}^{\left(l\right)}\sim Bernoulli\left(p\right) \end{array}$$

(8) $$\begin{array}{l} {\tilde{y}}^{\left(l\right)}=r^{\left(l\right)}•y^{\left(l\right)} \end{array}$$

(9) $$\begin{array}{l} z_{i}^{\left(l+\right)}=w_{i}^{\left(l+1\right)}{\tilde{y}}^{\left(l\right)}+b_{i}^{\left(l+1\right)} \end{array}$$

(10) $$\begin{array}{l} y_{i}^{\left(l+1\right)}=f\left(z_{i}^{\left(l+1\right)}\right) \end{array}$$

The function of the Bernoulli function above is to randomly generate a vector with the probability coefficient *p* (value 0 or 1), indicating whether each neuron needs to be discarded. If the value is 0, the neuron does not calculate gradients or participate in subsequent error propagation. In this paper, we used a 50% dropout to reduce overfitting. Spatial dropout is implemented after the convolutional layer. Deleting the entire feature map rather than a single element helps improve the independence between feature maps.

The essence of the neural network training process is the learning data distribution. If the distribution of the training data and the test data is different, it will greatly reduce the generalization ability of the network. Therefore, we need to normalize all input data before the training starts.

The batch normalization (Dose et al., 2018; Wang J. et al., 2019) method is for each batch of data, adding normalization processing (mean value is 0, standard deviation is 1) before each layer of the network input. That is, for any neuron in this layer (assuming the k-th dimension), ${\hat{x}}^{\left(k\right)}$ uses the following formula:

(11) $$\begin{array}{l} {\hat{x}}^{\left(k\right)}=\frac{x^{\left(k\right)}-E\left[x^{\left(k\right)}\right]}{\sqrt{Var\left[x^{\left(k\right)}\right]}} \end{array}$$

where *x*(*k*) is the original input data of the kth neuron in this layer, *E*[*x*(*k*)] is the mean of the input data in the kth neuron, and $\sqrt{Var\left[x^{\left(k\right)}\right]}$ is the standard deviation of the data in the kth neuron.

Batch normalization imposes additional constraints on the distribution of the data, which enhances the generalization ability of the model. The input distribution after normalization is forced to 0 mean and 1 standard deviation. To restore the original data distribution, transformation reconstruction, and learnable parameters γ and β are introduced in the specific implementation:

(12) $$\begin{array}{l} y^{\left(k\right)}=\gamma^{\left(k\right)}{\hat{x}}^{\left(k\right)}+\beta^{\left(k\right)} \end{array}$$

where γ^(*k*)^ and β^(*k*)^ are the variance and deviation of the input data distribution, respectively. In the batch normalization operation, γ and β become the learning parameters of this layer, which are decoupled from the parameters of the previous network layer. Therefore, it is more conducive to the optimization process and improves the generalization ability of the model. The formula of the complete forward normalization process of the batch normalized network layer is as follows:

(13) $$\begin{array}{l} \mu =\frac{1}{N}\overset{N}{\underset{i=1}{\sum }}X_{i}^{\prime } \end{array}$$

(14) $$\begin{array}{l} \sigma^{2}=\frac{1}{N}\overset{N}{\underset{i=1}{\sum }}{\left(X_{i}^{\prime }-\mu \right)}^{2} \end{array}$$

(15) $$\begin{array}{l} X_{i}^{norm}=\frac{X_{i}^{\prime }-\mu }{\sqrt{\sigma^{2}+\varepsilon }} \end{array}$$

(16) $$\begin{array}{l} \tilde{X_{i}}=\gamma X_{i}^{norm}+\beta \end{array}$$

In this paper, the global averaged accuracy and ROC curve were used to evaluate the classification model. The global averaged accuracy is the ratio of the number of correctly classified samples to the total number of samples. The area under the ROC curve is expressed in AUC and ranges from 0.5 to 1. The closer the AUC is to 1.0, the higher the authenticity of the method. The performance of the model on the recognition of four types of MI was measured by precision, recall and F-score. The larger the values, the better the performance of the model. Here, TP, true positives; TN, true negatives; FP, false positives; FN, false negatives.

(17) $$\begin{array}{l} Global\;Average\;Accuracy=\frac{TP+TN}{TP+TN+FP+FN} \end{array}$$

(18) $$\begin{array}{l} Precision=\frac{TP}{TP+FP} \end{array}$$

(19) $$\begin{array}{l} Recall=\frac{TP}{TP+FN} \end{array}$$

(20) $$\begin{array}{l} F_{-score}=\frac{2\times Precision\times Recall}{Precision+Recall}. \end{array}$$

## 3. Results

In this paper, 10-fold cross validation was carried out for the dataset. Ninety percent of the dataset was used as the training set for training the CNN model to verify its robustness to data changes. Ten percent of the dataset was used as the test set to verify the validity of the model. The training set and test set were normalized, and then sent to CNN for operation.

### 3.1. Accuracy of Electrode Pairs

On 10 subjects dataset, we conducted 9 groups of single pair experiments to test their global averaged accuracy and the accuracy of four MI tasks (T1, T2, T3, T4). Each group was tested 10 times and then averaged. The average value is taken as the global average accuracy of the electrode pair, as shown in Table 2. The global averaged accuracy of the test set is the average accuracy of 9 electrode pairs.

**Table 2:** Accuracy of electrode pairs.

**10 subjects**	**Global averaged accuracy (%)**	**T1 accuracy (%)**	**T2 accuracy (%)**	**T3 accuracy (%)**	**T4 accuracy (%)**
Training set	100	100	100	100	100
Test set	95.83	93.66	97.35	98.30	92.44
FC1-FC2	95.44	97.12	96.41	94.47	94.04
FC3-FC4	98.61	99.00	97.27	98.03	100.00
FC5-FC6	88.73	94.86	94.76	94.69	76.80
C1-C2	98.35	98.32	97.02	98.82	100.00
C3-C4	97.09	96.05	96.31	96.26	100.00
C5-C6	91.77	97.70	89.73	96.18	83.57
CP1-CP2	97.59	96.18	95.22	99.11	100.00
CP3-CP4	96.20	93.73	93.53	97.97	100.00
CP5-CP6	90.00	90.19	94.82	95.79	79.43

In Table 2, the upper 2 rows are the accuracies of training set and test set, and the following 9 rows are the results of each pair of electrodes. The highest global averaged accuracy of single pair was FC3-FC4, reaching 98.61%, and the accuracies of four MI tasks are also relatively high, at 99.00, 97.27, 98.03, and 100.00%, respectively. FC5-FC6 has the lowest global averaged accuracy of 88.73%, and four MI accuracies are 94.86, 94.76, 94.69, and 76.80%, respectively. The first three classification accuracies are relatively high, and the effect of T4 (both feet) MI task is general.

### 3.2. Accuracy Within Individual Subjects

To obtain the global averaged accuracy within individual subjects, we divided all trials of a specific subject into 10 parts, nine for training and one for testing. This ensured that no blocks of data are split across training and test sets.

Then we trained and tested the model to get the accuracy. From data segmentation to training and testing, we made 10 cycles for each subject. Their average is taken as the global averaged accuracy of individual subject. The accuracies are as follows: S1 (93.08%), S2 (95.71%), S3 (95.42%), S4 (96.56%), S5 (94.61%), S6 (95.25%), S7 (96.82%), S8 (94.86%), S9 (95.81%), S10 (96.02%).

**Figure 4:**
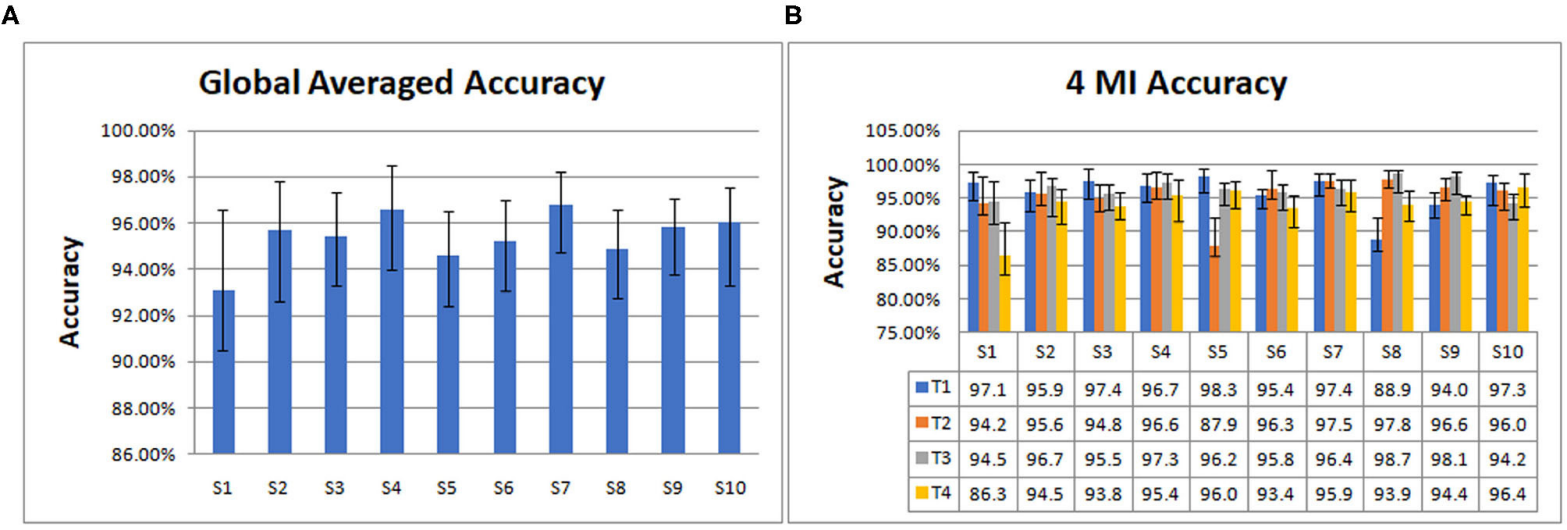
The global averaged accuracy of an individual subject on 10 subjects dataset. **(A)** Column chart of the global averaged accuracy of an individual subject. **(B)** Column chart of four types of MI accuracy (left fist, right fist, both fists and both feet, which are called T1, T2, T3, and T4) of an individual subject.

From Figure 4A, it can be seen that S7 has the highest global average accuracy, and S1 has the lowest accuracy. The four MI accuracies of an individual subject are shown in Figure 4B, T1 achieves the highest accuracy on S5, T2 on S8, T3 on S8, T4 on S10. In addition, T1 has the lowest accuracy on S8, T2 on S5, T3 on S10, and T4 on S1.

### 3.3. Classification on Different Dataset

Our proposed method has also been trained and evaluated on different amounts of participants. Ten subjects (7,560 samples), 20 subjects (15,120 samples), 60 subjects (45,360 samples), and 100 subjects (75,600 samples) from the Physionet dataset were used.

**Figure 5:**
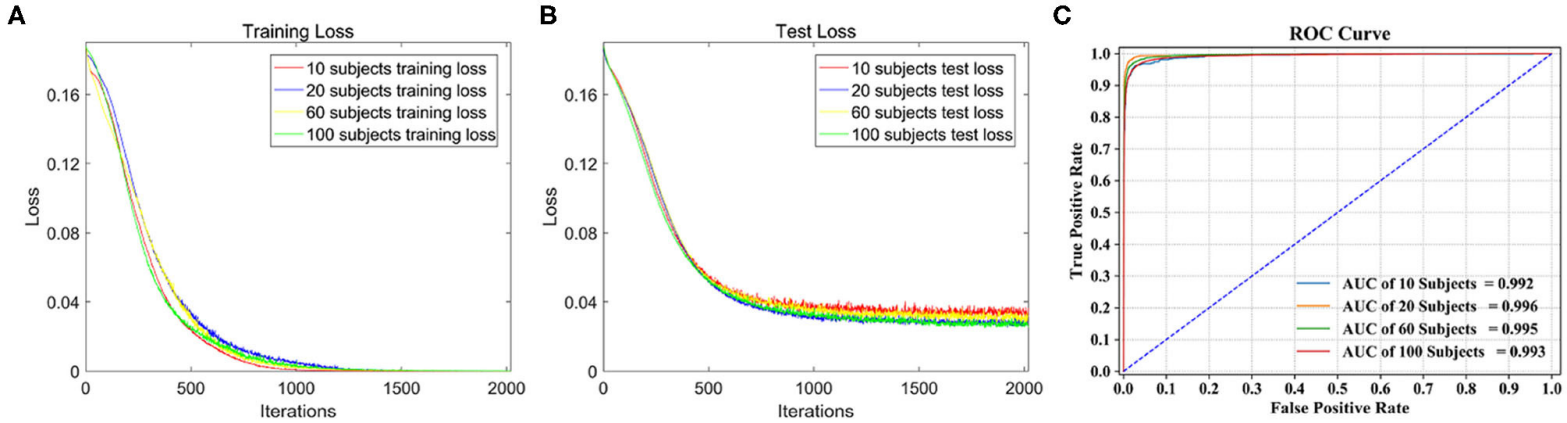
Classification performance comparison on different dataset. **(A)** The loss function curve of different subjects on the training set. **(B)** The loss function curve of different subjects on the test set. **(C)** ROC curve and AUC of the four test sets.

The loss function curves of different subjects are detailed in Figure 5. We can observe the convergence of the models under different subjects. The abscissa represents the number of iterations, and the ordinate represents the loss value. Figure 5A shows the loss curves of the training set. From the comparison of four loss curves, it can be observed that the loss value decreases with the increase in iterations, and then remains basically stable to achieve the best training effect of the model. At this time, their training losses are almost 0, and the trained models are the optimal classification models. Figure 5B shows the loss curves of the test set on the optimal models, which decrease to about 0.04 as the number of iterations increase. Therefore, the models are convergent in training set and test set.

Four types of dataset were used for model training, and four classification models were obtained. Table 3 shows the global average accuracy of CNN models in different datasets. The accuracies of all training sets are 100%, and the accuracies of four test sets are different. Among them, the accuracy of 20 subjects is 97.28%, and the corresponding model has the best classification performance.

**Table 3:** The global average accuracy of the CNN model in different dataset.

**Accuracy**	**10 subjects (%)**	**20 subjects (%)**	**60 subjects (%)**	**100 subjects (%)**
Training set	100	100	100	100
Test set	95.76	97.28	96.01	94.80

The ROC curve is given in Figure 5C, AUC of 10, 60, and 100 subjects are 0.992, 0.995, and 0.993, respectively, and the AUC of 20 subjects stands out at 0.996, so its corresponding model classification performance is the best.

**Figure 6:**
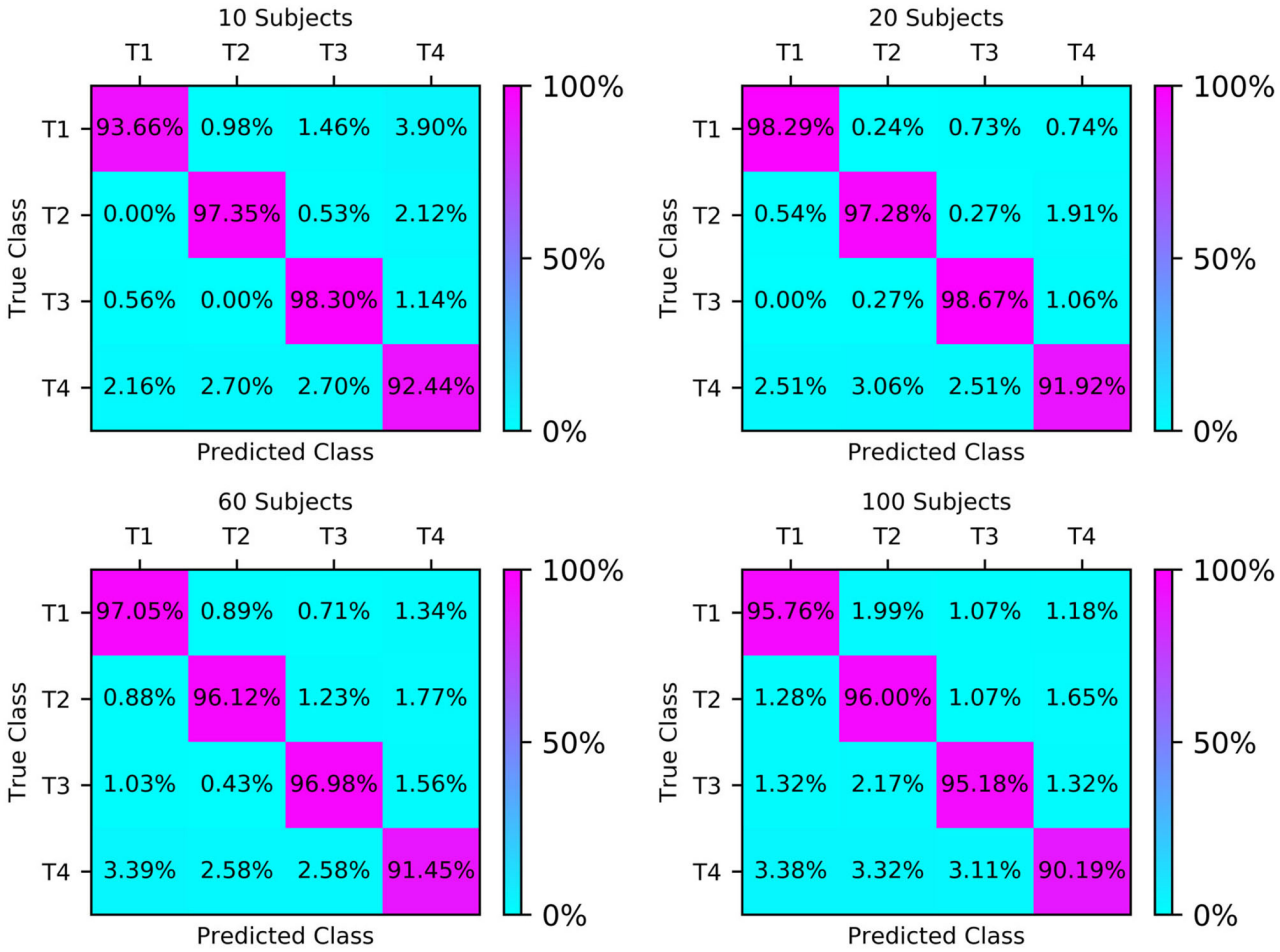
Classification accuracy confusion matrix of the four types of MI (T1, T2, T3, T4) on different test sets.

The confusion matrices of the four test sets illustrate their group-level classification results, as shown in Figure 6. The numbers in the diagonal lines represent the percentage of correct classification, and the other numbers represent the percentage of misclassification. The results showed that the confusion matrix of 20 subjects performed best, with the correct classification rates for T1, T2, T3, and T4 being 98.29, 97.28, 98.67, and 91.92%, respectively.

**Figure 7:**
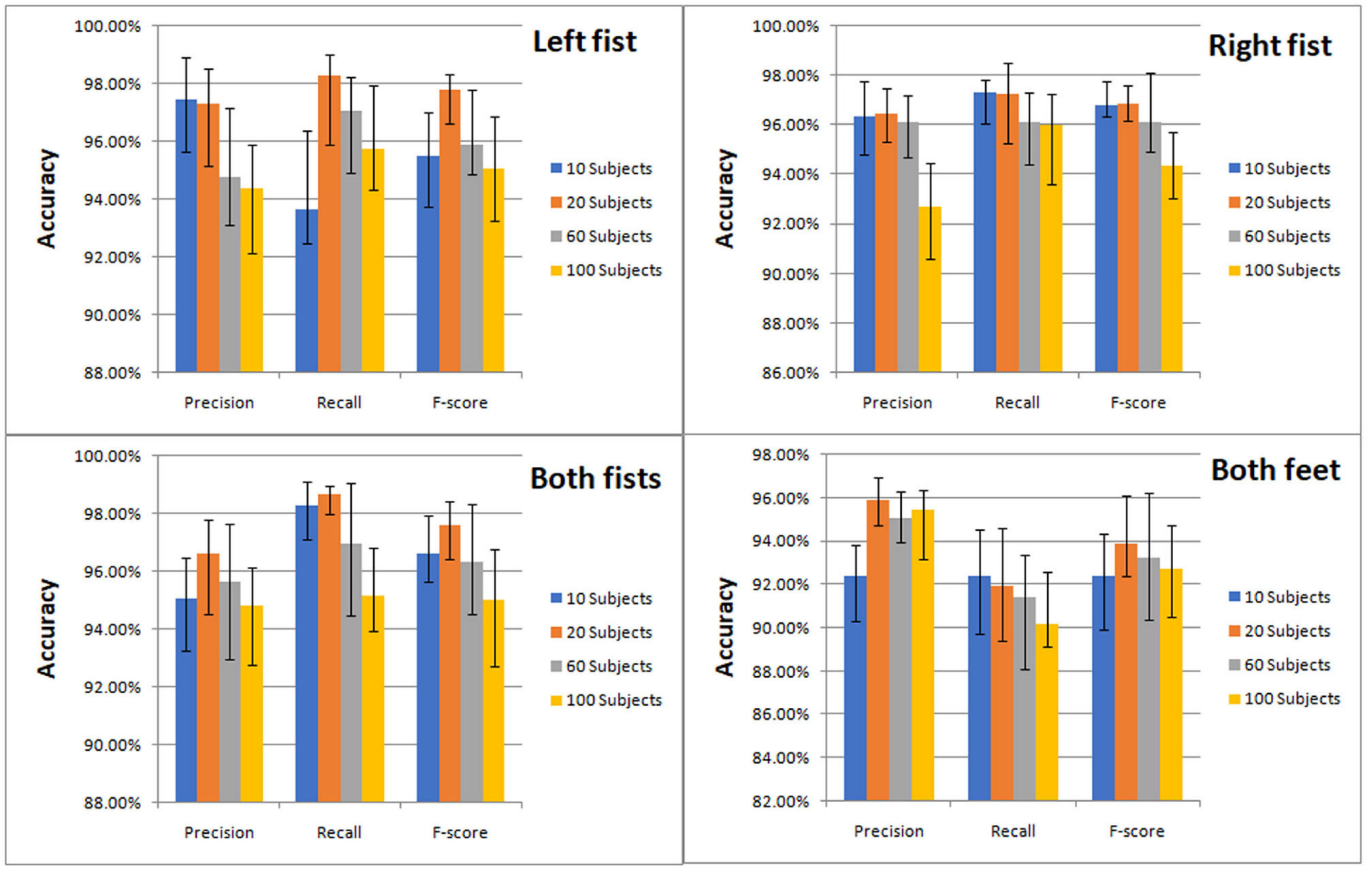
Classification accuracy column chart of the four types of MI (left fist, right fist, both fists, and both feet) on different test sets.

The classification results of the four types of MI by CNN were measured by precision, recall and F-score. We compared the classification effect of different test sets on left fist, right fist, both fists, and both feet. At a glance of Figure 7, we can find that the models of the different test sets have achieved good classification performance.

To show the quantitative results for using the models on subjects not included in the training sets, we conducted the relevant experiments on the different dataset, respectively. We selected the data of subjects who had never participated in the training, such as the data of subjects *S*_101_, *S*_105_, and *S*_109_ as the test set. The test accuracy for this subject-independent case is given in Table 4. The highest test accuracy is 73.80% achieved by *S*_101_ on the model of 100 subjects dataset, and the lowest test accuracy is 63.84% achieved by *S*_105_ on the model of 10 subjects dataset. For a single subject, we can see that better classification performance can be obtained with larger training datasets.

**Table 4:** The test accuracy of subject-independent on the model of the different dataset.

**No. of subject**	**10 subjects (%)**	**20 subjects (%)**	**60 subjects (%)**	**100 subjects (%)**
101	65.23	67.45	71.33	73.80
105	63.84	65.01	69.98	71.10
109	66.45	67.89	71.49	72.51

## 4. Discussion

### 4.1. Electrode Pair Accuracy Comparison

On 10 subjects dataset, we carried out 9 groups of single pair experiments to test their global average accuracy. The experiments use 10-fold cross validation, each group is tested 10 times, and then the average value is taken as the global average accuracy of each pair.

The placements of the electrode pairs is shown in Figure 2A, two electrodes of each pair are symmetrical to the Z sagittal line (Fpz-Cz-Iz). From the global average accuracy of each pair shown in Table 2, it can be roughly inferred that the accuracy of the electrode pair on the C coronal line is higher than that on the CP coronal line and the FC coronal line. Moreover, the closer the electrode pair is to the Z sagittal line, the higher the accuracy, and vice versa. Due to the physiological and psychological differences between individuals, the spatial origin and amplitude change of brain signals show specific patterns, which will cause high individual variability. It will affect the performance of the model and the electrode pairs to varying degrees. Therefore, the accuracy of each pair cannot fully meet certain rules. As shown in Table 2, the accuracy of FC3-FC4 is higher than that of FC1-FC2 and C3-C4. In the design of the BCI systems, a large number of experiments should be carried out according to the database established by users to avoid selecting electrode pairs with low accuracy as far as possible, which is also the focus of our next work.

### 4.2. Classification Comparison on Individual Subject

In this paper, 10-fold cross validation was carried out for the dataset. On the 10 subjects dataset, we conducted 10 groups of individual subject experiments. Each group of experiments has been cycled 10 times. We divided all trials of a specific subject into 10 parts on average, took one of them in turn for testing, and the rest for training. The average of 10 results is the global averaged accuracy, which reduces the randomness brought by data partitioning and helps to improve the stability of the model.

As indicated in Figure 4, the average accuracy of 10 subjects is 95.41%. S7 achieved the best classification effect, with the average accuracy of 96.83%. Its 4 MI accuracies are 97.4% (T1), 97.5% (T2), 96.4% (T3), and 95.9% (T4), respectively. The average accuracy of S1 is the lowest at 93.08%, and the MI accuracies are 97.1% (T1), 94.2% (T2), 94.50% (T3), 86.3% (T4), respectively. The accuracy of T4 is the lowest, indicating that the classification effect of S1 on T4, that is, both feet is the worst. The average accuracies of 10 subjects on 4 MI are 95.88% (T1), 95.36% (T2), 96.36% (T3), 94.05% (T4). Among the four types of MI tasks, the best is both fists and the worst is both feet.

### 4.3. Model Comparison

In the CNN model construction based on the dataset of 20 subjects, we used spatial dropout and BN to reduce the risk of overfitting. Table 5 shows the accuracy comparison of the CNN models under anti-overfitting measures, including global averaged accuracy and the accuracy of each of the four tasks. The data analysis is described in detail later.

**Table 5:** Comparison of accuracy of the CNN models under anti-overfitting measures.

**Model**	**Global averaged accuracy (%)**	**T1 accuracy (%)**	**T2 accuracy (%)**	**T3 accuracy (%)**	**T4 accuracy (%)**
CNN	97.28	98.78	97.28	98.13	94.71
CNN without dropout	95.30	97.07	94.57	96.27	93.04
CNN without BN	89.74	89.73	90.49	89.87	88.86
CNN without dropout BN	83.92	82.89	83.15	86.67	83.01

**Figure 8:**
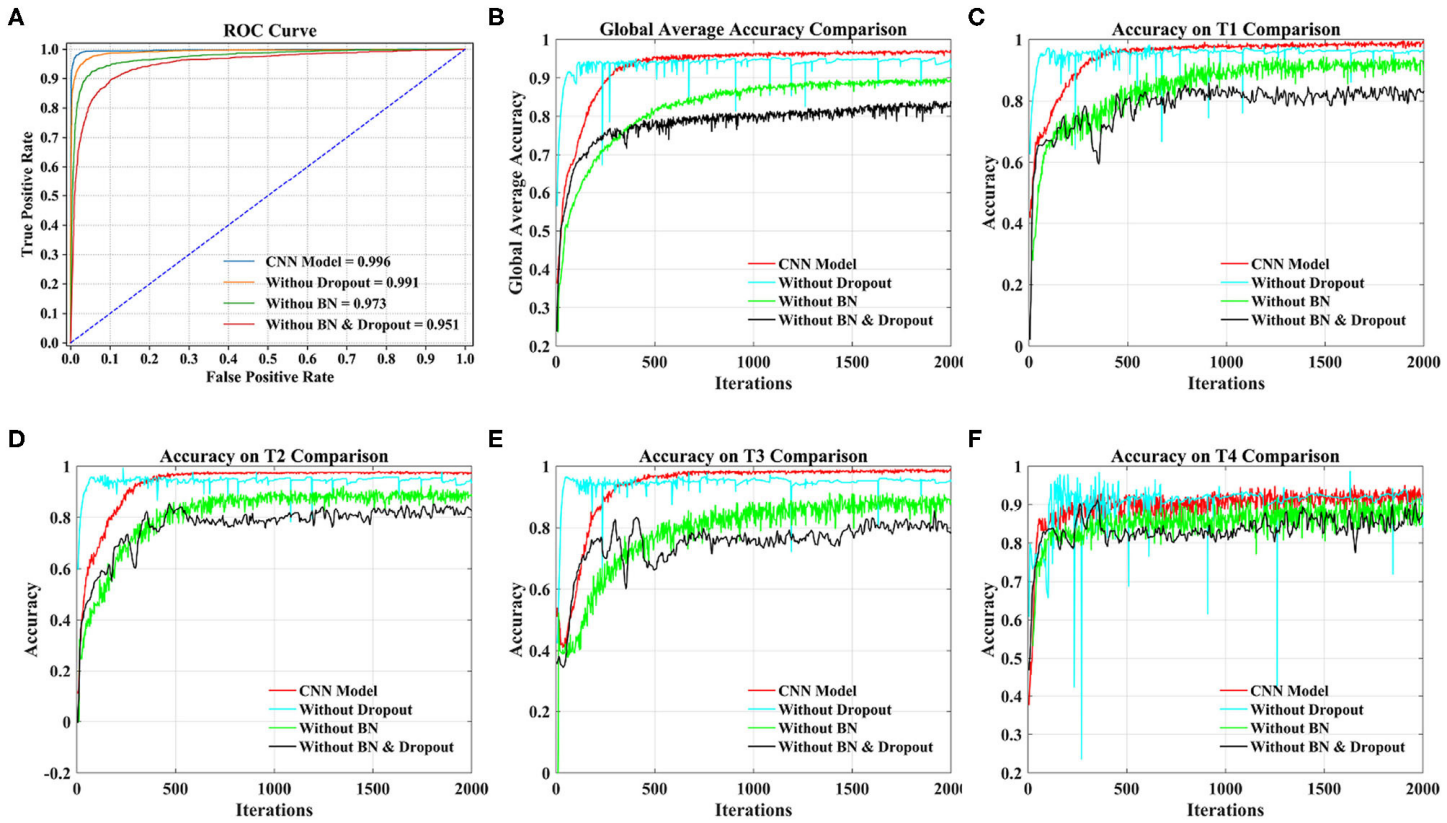
Comparison of different models under 20 subjects dataset. **(A)** Comparison of ROC curve and AUC of different models. **(B)** Comparison of the global average accuracy of different models. **(C)** Comparison of accuracy on T1 (left fist) of different models. **(D)** Comparison of accuracy on T2 (right fist) of different models. **(E)** Comparison of accuracy on T3 (both fists) of different models. **(F)** Comparison of accuracy on T4 (both feet) of different models.

From Figure 8A, we can see that the AUC of the CNN model stands out at 0.996, followed by 0.991 without dropout, 0.973 without BN, and 0.951 without dropout and BN.

According to Figure 8B, the order of the models to reach the steady state from fast to slow is the model without dropout, our proposed CNN model, the model without dropout and BN, and the model without BN. The curve of our proposed model reached a stable state after 500 iterations and achieved the highest accuracy. At this time, the accuracy of the model without dropout and BN and the model without BN are still slowly increasing. The model without dropout reached a stable state as soon as possible. However, the model is prone to overfitting without dropout operation, resulting in sharp curve burr and unstable performance during the whole iteration. By observing the smoothness of the four curves, our proposed model has the smoothest curve, the least furr and the most stable performance. Then, we refer to the values in Table 5 to compare the global average accuracy. In the final stable state, the accuracy of our proposed model is the highest at 97.28%, followed by 95.30% without dropout, 89.74% without BN, and 83.92% without dropout and BN. The proposed model is 1.98% higher than the model without dropout, 7.54% higher than the model without BN, and 13.36% higher than the model without dropout and BN.

Figures 8C–F illustrate the accuracy of our proposed model on the four tasks in detail. The growth trend and performance of T1, T2, and T3 curves are similar to those in Figure 8B. The main difference is the poor performance of the T4 task, that is, both feet. Even the proposed model has uneven curves throughout the iteration, and fluctuates above and below a certain value. The curve performance of the other models is worse, especially the model without dropout. This will be the focus of our future research. With reference to the values in Table 5, the accuracy of our proposed model on four tasks (T1, T2, T3, T4) reached the peak at 98.78, 97.28, 98.13, and 94.71%, respectively. Our model is 1.71% (T1), 2.71% (T2), 1.86% (T3), and 1.67% (T4) higher than the model without dropout, 9.05% (T1), 6.79% (T2), 8.26% (T3), and 5.85% (T4) higher than the model without BN, and 15.89% (T1), 14.13% (T2), 11.46% (T3), and 11.70% (T4) higher than the model without dropout and BN.

Taking the 20 subjects dataset as an example, we compared our proposed CNN model, the model without dropout, the model without BN and the model without dropout and BN from the ROC curve, the global average accuracy curve and the accuracy curve of four MI tasks. The experimental results illustrate that our proposed CNN model has the smoothest curve, the least furr, the most stable performance. In general, the use of spatial dropout and BN in CNN can effectively reduce the risk of overfitting and improve the generalization ability and classification effect of the model.

### 4.4. Classification Comparison

Based on the same database and the same number of MI tasks, our proposed method has been compared with other works in Table 6. We can observe that CNN method is indeed effective in MI classification, which can greatly improve the classification accuracy.

**Table 6:** Results comparison on Physionet database.

**Work**	**Number of electrodes**	**Number of MI tasks**	**Database**	**Global averaged accuracy (%)**	**Methods**
Kim et al. (2016)	14	4	Physionet	77.70	SUTCCSP
Pinheiro et al. (2018)	2	4	Physionet	74.96	RNA
Dose et al. (2018)	64	4	Physionet	80.38	CNN
Karácsony et al. (2019)	64	4	Physionet	76.37	CNN
Hou et al. (2019)	–	4	Physionet	95.54	ESI+CNN
This work	2	4	Physionet	97.28	CNN

In the same method, compared with Dose et al. (2018) and Karácsony et al. (2019), our work achieved superior performance, and used two electrodes to greatly reduce the sample size and data dimensions.

Although, Hou et al. (2019) used multiple electrodes whereas this work used only two electrodes. Hou et al. used the Colin27 template brain for Physionet database, the boundary element method (BEM) implemented in the OpenMEEG toolbox for a realistic-geometry head model, and a Morlet wavelet approach utilized for feature extraction. Its preprocessing process is very complicated. However, this work is based on the original data as the input of CNN, without any preprocessing or artificial feature extraction operations. Therefore, this work can simplify the BCI design. Moreover, Hou et al. mainly used data sets of 10 subjects with an accuracy of 94.54%, while our work achieved 95.76, 97.28, 96.01, and 94.8% on the data sets of 10, 20, 60, and 100 subjects, respectively. So this work has larger data sets and higher global average accuracy.

## 5. Conclusion

In this paper, we proposed a CNN architecture and design the network structure and parameters. Without any preprocessing and artificial feature extraction operations, our model can classify the raw MI-EEG signals of the left and right brain symmetric electrodes.

Using the Physionet database as the data source, the model was trained and tested on 10, 20, 60, and 100 subjects, respectively. The experimental results indicate that our models are convergent on both the training set and the test set. It can reach the uppermost accuracy on group-level classification, with 95.76% accuracy for 10 subjects, 97.28% for 20 subjects, 96.01% for 60 subjects, and 94.80% for 100 subjects. In addition, the global average accuracy of T1, T2, T3, and T4 on 20 subjects dataset can reach 98.29, 97.28, 98.67, and 91.92%, respectively. The electrode pair with the highest global averaged accuracy on 10 subjects dataset is FC3-FC4, with 98.61%. Our proposed approach manages to decode MI-EEG raw signals with yielding remarkable robustness and adaptability, simplify the BCI systems design and pave the road to build practical clinical applications.

In future work, we will build a real-time EEG signals acquisition system, and use self-built database to verify the validity and robustness of the proposed method.

## Data Availability Statement

All datasets presented in this study are included in the article/supplementary material.

## Author Contributions

XL was responsible for neural network design and paper writing. ZY was in charge of the design of the overall framework of the paper. TC was in charge of reference reading. FW was in charge of data processing. YH was responsible for the accuracy of the grammar of the article. All authors contributed to the article and approved the submitted version.

## Conflict of Interest

The authors declare that the research was conducted in the absence of any commercial or financial relationships that could be construed as a potential conflict of interest.
